# Screening of wild deer populations for exposure to SARS‐CoV‐2 in the United Kingdom, 2020–2021

**DOI:** 10.1111/tbed.14534

**Published:** 2022-04-08

**Authors:** Maya Holding, Ashley David Otter, Stuart Dowall, Katsuhisa Takumi, Bethany Hicks, Tom Coleman, Georgia Hemingway, Matthew Royds, Stephen Findlay‐Wilson, Mollie Curran‐French, Richard Vipond, Hein Sprong, Roger Hewson

**Affiliations:** ^1^ Virology and Pathogenesis Group UK Health Security Agency, Porton Down Salisbury UK; ^2^ Health Protection Research Unit in Emerging and Zoonotic Infections National Institute for Health Research Liverpool UK; ^3^ SARS‐CoV‐2 Serosurveillance Laboratory UK Health Security Agency, Porton Down Salisbury UK; ^4^ Centre for Infectious Disease Control National Institute for Public Health and the Environment Bilthoven the Netherlands; ^5^ Faculty of Infectious and Tropical Diseases London School of Hygiene and Tropical Medicine Keppel Street London UK

**Keywords:** COVID‐19 serological testing, deer, SARS‐CoV‐2, sentinel surveillance, United Kingdom, viral zoonoses

## Abstract

Following findings in Northern America of SARS‐CoV‐2 infections in white‐tailed deer, there is concern of similar infections in European deer and their potential as reservoirs of SARS‐CoV‐2 including opportunities for the emergence of new variants. UK deer sera were collected in 2020–2021 from 6 species and a hybrid with 1748 tested using anti‐spike and anti‐nucleocapsid serology assays. No samples were positive on both assays nor by surrogate neutralization testing. There is no evidence that spill‐over infections of SARS‐CoV‐2 occurred from the human population to UK deer or that SARS‐CoV‐2 has been circulating in UK deer (over the study period). Although it cannot be ruled out, study results indicate that spill‐over infections followed by circulation of SARS‐CoV‐2 to the most common European deer species is small.

## 1 INTRODUCTION

Evidence from Northern America shows the potential of white‐tailed deer (WTD, *Odocoileus virginianus*) as a SARS‐CoV‐2 reservoir. WTD fawns inoculated with SARS‐CoV‐2 have been shown to shed infectious virus up to 5 days post‐infection. This shedding has been shown to be transmissible to unchallenged contact deer, resulting in seroconversion and the development of neutralizing antibodies (Martins et al., 2021; Palmer et al., 2021). In addition, SARS‐CoV‐2 RNA was detected in 36% of free‐ranging WTD collected from multiple locations within the state of Ohio during January–March 2021, including evidence of sustained transmission within this deer population (Hale et al., 2021). Furthermore, three different SARS‐CoV‐2 lineages genetically similar to human viruses were detected indicating that multiple reverse zoonosis events are likely to have occurred (Hale et al., 2021). SARS‐CoV‐2 RNA was also detected in WTD surveyed in Québec, Canada, albeit a lower proportion, with 1.2% of nasal swabs positive (Kotwa et al., 2022). Wider scale exposure in free‐ ranging WTD across other areas in the United States has also been observed. For example two serosurveillance studies in 2021 of 385 samples collected in Texas (Chandler et al., 2021) and 54 samples collected from Michigan, Pennsylvania, Illinois, New Jersey and New York (Palermo et al., 2021), identified similar seroprevalences of 40% and 37% respectively. Both studies detected neutralizing antibodies based on a surrogate virus neutralization test (sVNT). Evidence of deer‐to‐deer transmission was also demonstrated in a captive cervid facility in Texas, where 94.4% of WTD sampled were found to be seropositive by neutralization assay. Two other facilities sampled as part of the study found no evidence of exposure (Roundy et al., 2022). Informed by these findings, the World Organisation for Animal Health is recommending monitoring cervids in all regions, to increase knowledge and understanding of the likelihood of SARS‐CoV‐2 infection and circulation in other deer populations (World Organisation for Animal Health, 2021). Here, we refer to ‘exposure’ as (past) infection which has given rise to an immune response. Understanding the potential exposure of European deer species to SARS‐CoV‐2 and whether they could act as a reservoir is important, since these animals could become a source of new infections to other wildlife species, providing new routes for SARS‐CoV‐2 to evolve and resulting in new transmission opportunities of novel variants. Moreover, given the known plasticity of the SARS‐CoV‐2 genome (McCormick et al., 2021), new variants especially from animal reservoirs may not be well recognized by human immune systems.

In the United Kingdom, it is currently unknown whether deer have become infected with SARS‐CoV‐2. Different deer species are present in Europe to those in Northern America, with distinctive ecological and behavioural traits. In addition, human factors such as population density, behaviour and infrastructure are different, influencing the risk of exposure at the human–wildlife interface. The United Kingdom has a relatively high diversity of species including significant human interactions with deer populations. The United Kingdom also has one of the highest human and deer population densities in Europe (Burbaite & Csányi, 2009, 2010; Eurostat, n.d.).

Scientists at the UK Health Security Agency (UKHSA) have been using the UK deer population as sentinels for disease surveillance for several years, identifying the emergence of tick‐borne encephalitis virus in the United Kingdom via this route in 2019 (Holding et al., 2020). For SARS‐CoV‐2 three potential outcomes could result from a serosurveillance study in deer: (i) deer are not exposed and therefore no antibody response is detected; (ii) exposure which results in production of an antibody response, but there is no or only low‐level transmission between deer; or (iii) exposure, onward transmission and established circulation of SARS‐CoV‐2 within the deer population.

## 2 MATERIALS AND METHODS

Deer serum samples were collected by volunteers from routine culling operations in the United Kingdom between January 2020 and May 2021 previously as described (Holding et al., 2020). Blood was sampled from pooled blood within the chest cavity during gralloching; samples were taken as soon as possible after the deer were culled. Ethical approval was granted for the collection of these samples by the Public Health England Research Ethics and Governance of Public Health Practice Group on 10 October 2019. A total of 1748 serum samples were collected and tested, of which 654 were collected from January to the end of March 2020, during which point D614G was the main circulating variant. A total of 1094 samples were collected from October 2020 to May 2021 when Alpha predominated, Delta started to appear in March 2021 but only became the predominate strain in May 2021 (Public Health England, 2021). These time periods cover the open main open seasons for culling deer for the duration of this study. Samples were collected from all UK deer species (Table 1), the sample set is in general reflective of the overall abundance of deer species in the United Kingdom (Mathew et al., 2018). Both sexes of deer were included, with 1157 female and 567 male serum (24 unspecified) samples tested.

**TABLE 1:** Roche Elecsys® anti‐SARS‐CoV‐2 S and N assay result and GenScript SARS‐CoV‐2 surrogate virus neutralization test (sVNT) result

	S Elecsys® anti‐SARS‐CoV‐2		N Elecsys® anti‐SARS‐CoV‐2		Surrogate VNT*
Deer species	Positive/tested (%)	95% CI**	Positive/tested (%)	95% CI**	Positive/tested (%)
Chinese Water (*Hydropotes inermis*)	0/3 (0.0)	0.0–70.8	0/3 (0.0)	0.0–70.8	N/A
Fallow (*Dama dama*)	7/563 (1.2)	0.5–2.5	3/563 (0.5)	0.1–1.5	0/10 (0.0)
Muntjac (*Muntiacus reevesi)*	2/153 (1.3)	0.2–4.6	0/153 (0.0)	0.0–2.4	0/2 (0.0)
Red (*Cervus elaphus*)	17/436 (3.9)	2.3–6.2	3/436 (0.7)	0.1–2	0/20 (0.0)
Red/Sika hybrid	0/1 (0)	0.0–97.5	0/1 (0.0)	0.0–97.5	N/A
Roe (*Capreolus capreolus*)	28/555 (5)	3.4–7.2	3/555 (0.5)	0.1–1.6	0/31 (0.0)
Sika (*Cervus nippon*)	0/33 (0.0)	0.0–10.6	0/33 (0.0)	0.0–10.6	N/A
Unknown	0/4 (0.0)	0.0–60.2	0/4 (0.0)	0.0–60.2	N/A
Total	54/1748 (3.1)	2.3–4	9/1748 (0.5)	0.2–1	0/63 (0.0)

*Note*: The sVNT positive cut off value is ≥30% inhibition.

* Only S or N positive samples tested.

** The 95% confidence intervals were calculated using Fisher's exact test.

All samples were tested using the Roche Elecsys® Anti‐SARS‐CoV‐2 assay (Burgess Hill, UK), detecting total antibodies to the receptor binding domain (RBD) of spike (S) and also the nucleocapsid (N) as per manufacturer's instructions. Due to the assay being species‐independent with detection readouts based on antibodies binding to labelled antigens detected by electrochemiluminescence (ECL), it can be applied to detect antibodies from diverse species.

Any samples which were positive for either S or N antibodies were further tested using the GenScript cPass SARS‐CoV‐2 surrogate virus neutralization test (sVNT) (Oxford, UK), product code L00847‐C, according to the manufacturer's instructions. The criteria for indicating probable exposure to SARS‐CoV‐2 were assigned as either (i) samples which were positive on both the S and N assays or (ii) samples which were positive on either the S or N assay and also tested positive in the confirmatory sVNT.

## 3 RESULTS

**FIGURE 1:**
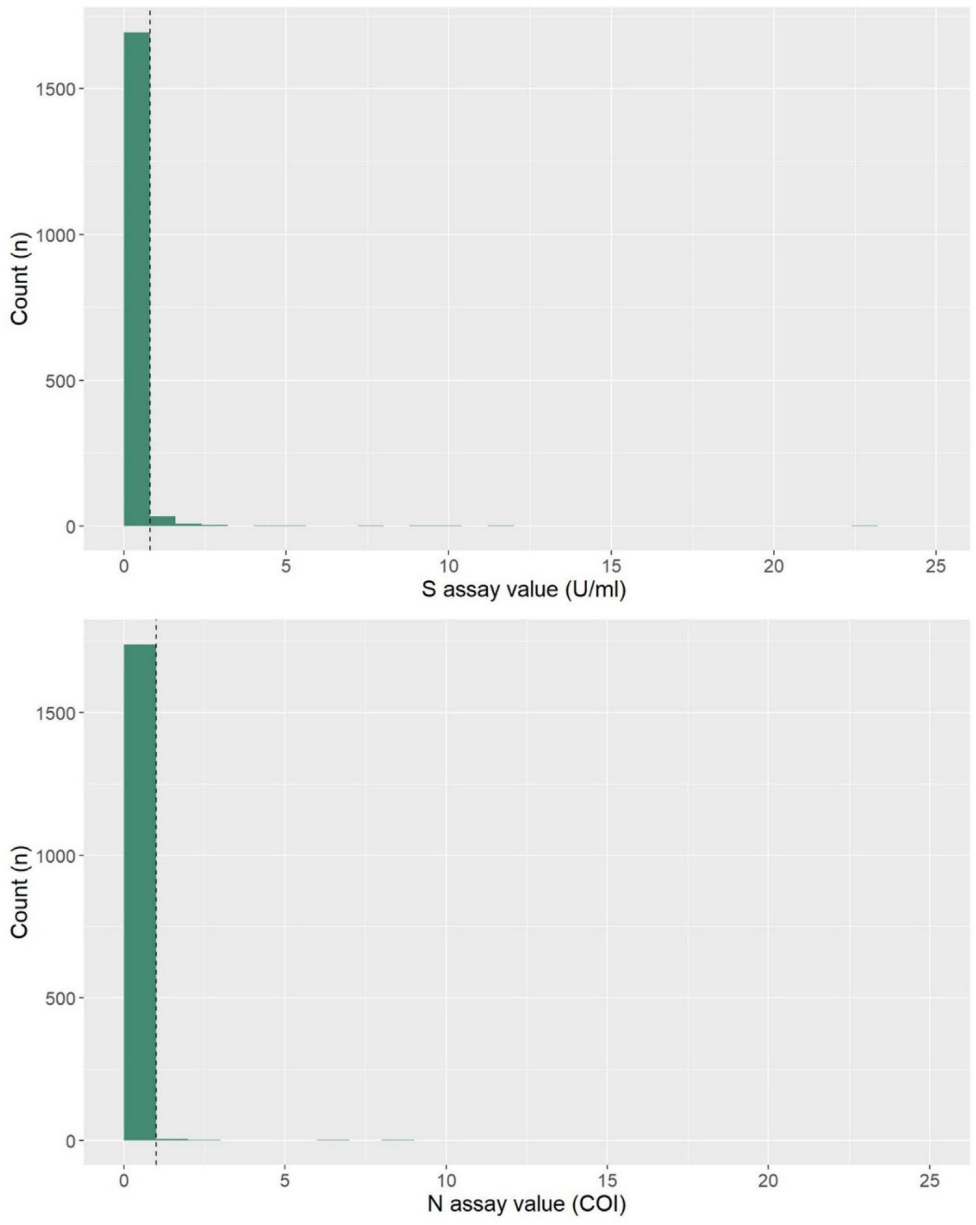
Distribution of S and N assay values; the dashed lines indicate the assay positive cut‐off values. The positive cut‐off values are >0.8 for the S assay and >1.0 for the N

Fifty‐four deer serum samples were positive on the S assay [positive cut‐off value = 0.8 units per ml (U/ml)], and 9 on the N assay [positive cut‐off value = 1.0 cut‐off index (COI)]. The majority (63.0%) of samples that were positive on the S assay were narrowly above the manufacturer's cut‐off (Figure 1), measuring between 0.8 and 1.6 U/ml. Only 20 samples measured above this. A negative binomial distribution was found for the assay values for both the S and N assays (Figure 1). None of the samples tested positive on both assays and none of the samples that were positive on either of the Elecsys® Anti‐SARS‐CoV‐2 assays were positive when tested with the sVNT. Therefore, none of the samples met the study criteria of sample seropositivity in both the S and N assays or one of these assays and positivity in sVNT, indicating no evidence of exposure and subsequent seroconversion to SARS‐CoV‐2 (Table 1).

**FIGURE 2:**
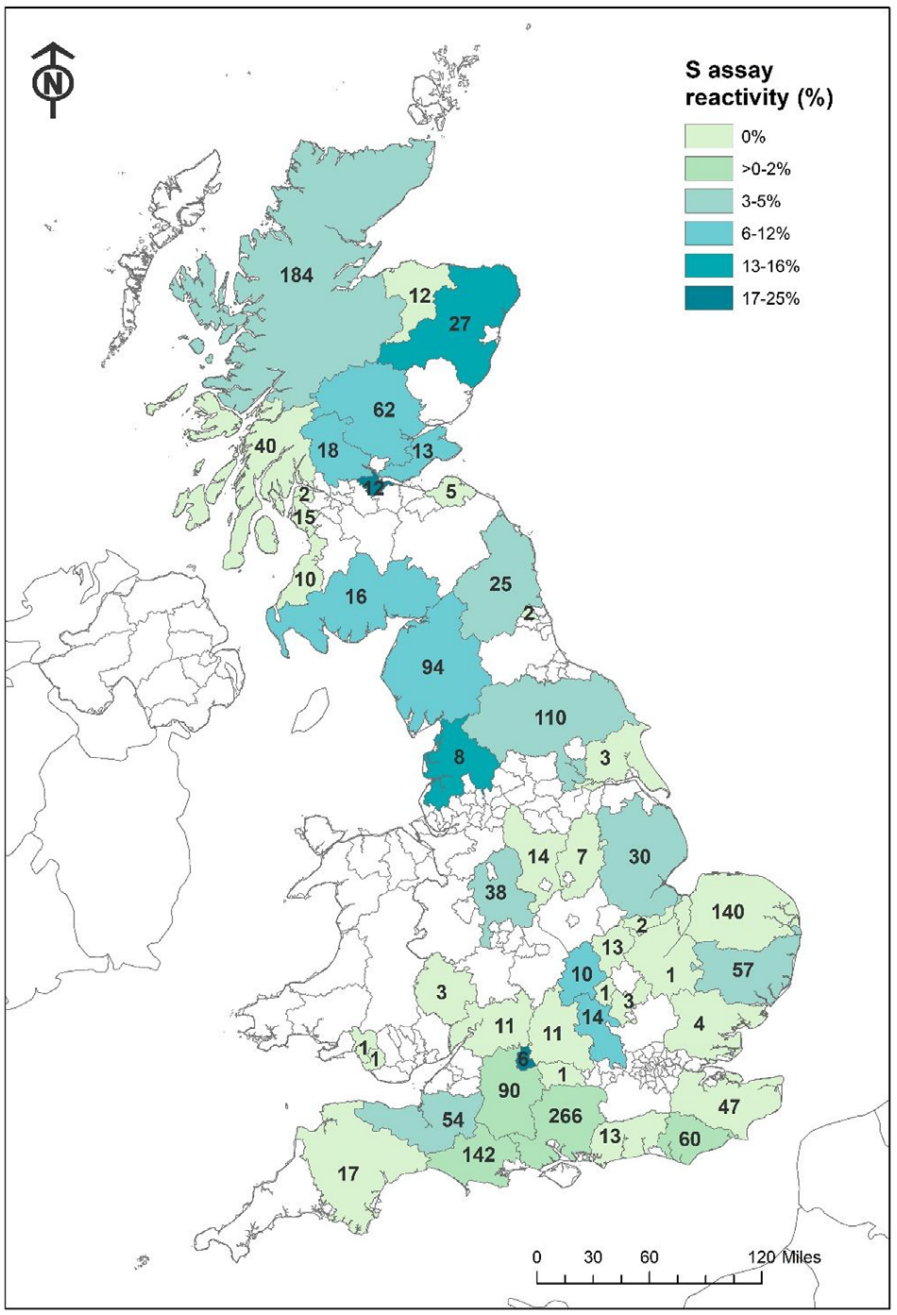
Distribution of samples by county/Unitary Authority; the number indicating the sample size of each and colour indicating percentage of samples appearing positive on the S assay. *Source*: Contains Ordnance Survey data © Crown copyright and database right (2021) and National Statistics data © Crown copyright and database right (2021). © Esri, DeLorme

The proportion of samples above the positive cut‐off value for the S assay was numerically the highest in roe deer (5.1%), followed by red deer (3.9%). Given that a reasonably representative sample was taken from the deer population in the United Kingdom, both red and roe deer contributed most to the apparent S antigen reactivity. This finding was supported by a binomial generalized linear model and logit link, in combination with a step algorithm and AIC criteria to identify the most parsimonious model (McCullagh & Nelder, 1983). Deer serum samples were unevenly collected from 48 counties/Unitary Authorities (Figure 2). These were generally collected from across England and Scotland, with only two Welsh counties, with one sample from each. No samples were collected in Northern Ireland. High apparent S antigen reactivities were found in Falkirk (25.0%), Aberdeenshire (14.8%), Perth and Kinross (8.1%) and Cumbria (6.4%) (Figure 2). The reactivity in the N assay was negligibly low, between 0% and 0.7% for all deer species (Table 1).

## 4 DISCUSSION

Results from this study indicate there is no serological evidence of significant circulation of SARS‐CoV‐2 in UK deer over the study period and provide no evidence that the deer were exposed to SARS‐CoV‐2. There was no agreement between the S and N antigen assay results and no neutralizing antibodies to SARS‐CoV‐2 were detected. Furthermore, there was no evidence of a bimodal distribution, with either the S or N Elecsys® Anti‐SARS‐CoV‐2 assays, which would have been expected for a seropositive subpopulation in a serosurvey (Jacobson, 1998).

Our study findings provide a different picture to that found in WTD across Northern America, where two studies in different locations found high seroprevalences of 40% and 37%; each using the same sVNT as used in this study for confirmatory testing (Chandler et al., 2021; Palermo et al., 2021). While the deer in this study are of different species to the Northern American WTD, it is still possible that UK deer are permissive to SARS‐CoV‐2, since the angiotensin‐converting enzyme 2 (ACE2) receptor is present in all species of deer, though they may have varying sequence differences for the key residues for SARS‐CoV‐2 binding (Damas et al., 2020). The phylogenetic relationship to WTD of the UK deer species varies widely. Of the UK deer species, roe and CWD, are phylogenetically closest to WTD, both being part of the telemetacarpalian lineage, with the latter having a restricted distribution in the United Kingdom. The other deer species found in the United Kingdom, fallow, red, sika and muntjac are all more disparate from WTD, forming part of the Cervinae subfamily (Pitra et al., 2004). It may be that adequate opportunities for human‐to‐deer transmission have yet to be sufficiently established; the latest deer samples were taken in May 2021. The potential transmission route may be different in Europe as compared to Northern America, because of differences in human infrastructure and population distribution/densities or in ecological and behavioural traits between in UK deer species and WTD. Given that the ACE2 receptor is present in all species of deer, experimental infections with different European deer species or suitable in vitro models of them would provide useful confirmatory data for their susceptibility to SARS‐CoV‐2.

The most common deer species roe, red fallow and muntjac all had a sample size above what was calculated as required (≥139) for an assumed prevalence of 10% (Ausvet, 2022), indicating that if there were significant circulation of SARS‐CoV‐2 to the UK deer population over the study period, then it would have most likely been observed. Any undetected reverse zoonosis events are likely to have been of low level or only very recent introductions. To date, evidence of exposure and circulation in wider European deer species have not been assessed. This study suggests that currently, common wild European deer species are not supporting SARS‐CoV‐2 infections. The low levels of seroreactivity detected by the S and N antigen assays from red and roe deer samples in this study may suggest cross‐reactivity with related coronaviruses. Indeed a wide range of coronaviruses are known to circulate in wildlife, livestock and companion animals (Ghai et al., 2021). Livestock for example have been shown to be intermediate hosts in the emergence of three human coronaviruses and an unknown ungulate species, speculated to be cattle, is accepted as the intermediate host of human coronavirus OC43 (Vijgen et al., 2006, 2005). Many animal coronaviruses cause long‐term or persistent enzootic infections. Long periods of coronavirus infection combined with a high mutation rates increase the probability that a virus mutant with an extended host range may arise. Furthermore, given the promiscuous re‐combinatory ability of the coronaviruses, which are already known to contribute to their high zoonotic and pandemic potential (Forni et al., 2020; Pratelli et al., 2021), continued monitoring of the UK deer population, including other animal species (Forni et al., 2020; Maurin et al., 2021) would be sensible.

## CONFLICT OF INTEREST

The authors have declared no conflict of interest.

## ETHICS STATEMENT

The authors confirm that the ethical policies of the journal, as noted on the journal's author guidelines page, have been adhered to and the appropriate ethical review committee approval has been received for sample collection.

## Data Availability

The data that support the findings of this study are available on request from the corresponding author. The data are not publicly available due to privacy or ethical restrictions.
